# The Relation Between Official WhatsApp-Distributed COVID-19 News Exposure and Psychological Symptoms: Cross-Sectional Survey Study

**DOI:** 10.2196/22142

**Published:** 2020-09-25

**Authors:** Jean C J Liu, Eddie M W Tong

**Affiliations:** 1 Yale-NUS College Singapore Singapore; 2 Department of Psychology National University of Singapore Singapore Singapore

**Keywords:** mental health, social media, pandemic, depression, anxiety, stress, COVID-19, app, risk factor, psychology

## Abstract

**Background:**

In a global pandemic, digital technology offers innovative methods to disseminate public health messages. As an example, the messenger app WhatsApp was adopted by both the World Health Organization and government agencies to provide updates on the coronavirus disease (COVID-19). During a time when rumors and excessive news threaten psychological well-being, these services allow for rapid transmission of information and may boost resilience.

**Objective:**

In this study, we sought to accomplish the following: (1) assess well-being during the pandemic; (2) replicate prior findings linking exposure to COVID-19 news with psychological distress; and (3) examine whether subscription to an official WhatsApp channel can mitigate this risk.

**Methods:**

Across 8 weeks of the COVID-19 outbreak (March 7 to April 21, 2020), we conducted a survey of 1145 adults in Singapore. As the primary outcome measure, participants completed the Depression, Anxiety, and Stress Scale (DASS-21). As predictor variables, participants also answered questions pertaining to the following: (1) their exposure to COVID-19 news; (2) their use of the Singapore government’s WhatsApp channel; and (3) their demographics.

**Results:**

Within the sample, 7.9% of participants had severe or extremely severe symptoms on at least one DASS-21 subscale. Depression scores were associated with increased time spent receiving COVID-19 updates, whereas use of the official WhatsApp channel emerged as a protective factor (*b*=–0.07, *t*[863]=–2.04, *P*=.04). Similarly, increased anxiety scores were associated with increased exposure to both updates and rumors, but this risk was mitigated by trust in the government’s WhatsApp messages (*b*=–0.05, *t*[863]=–2.13, *P*=.03). Finally, although stress symptoms increased with the amount of time spent receiving updates, these symptoms were not significantly related to WhatsApp use.

**Conclusions:**

Our findings suggest that messenger apps may be an effective medium for disseminating pandemic-related information, allowing official agencies to reach a broad sector of the population rapidly. In turn, this use may promote public well-being amid an “infodemic.”

**Trial Registration:**

ClinicalTrials.gov NCT04305574; https://clinicaltrials.gov/ct2/show/NCT04305574

## Introduction

On March 11, 2020, the World Health Organization (WHO) declared that the outbreak of coronavirus disease (COVID-19) had become a global pandemic [1,2]. Within six months of the first reported case (in December 2019), more than 9 million people have been infected in over 200 countries and territories [3]. In response, an estimated 1 in 3 individuals worldwide have been placed under mandatory quarantines or movement restrictions as part of wide-ranging measures to control transmission [4].

Given the magnitude of the crisis, several studies have begun to document its impact on mental health [5]. For example, one study in China found that nearly all patients with COVID-19 (96%) had symptoms of posttraumatic stress disorder [6]. Within the general population, several studies have also recorded elevated levels of depression, anxiety, stress, and other psychological symptoms [2,7-9]. These findings underscore the need to address mental health as part of the broader public health response [10].

Preliminary research suggests that psychological symptoms during the outbreak are most common among those with preexisting health conditions [2], contacts of confirmed cases [2], and migrant and health workers [8,11]. Age, gender, and educational level have also been implicated as risk factors [7,8,11]. Although these demographic and situational characteristics allow vulnerable individuals to be identified, a separate set of risk factors may prove modifiable — those pertaining to the receipt of COVID-19 news.

To date, resilience during the pandemic has been observed when individuals receive regular updates on COVID-19 [2] and when they perceive the information to be adequate [2]. However, spending excessive time reading updates is associated with poorer outcomes [11-13]. Related to information availability, individuals also fare more poorly when they lack confidence in the health care infrastructure, and when they worry about potential infections among family members [2]. To the extent that these constitute modifiable risk factors, mitigation may be achieved when official government and transgovernmental bodies disseminate accurate and timely information, engendering trust in the management of COVID-19. This is particularly crucial in an age of social media, where false information has spread so rapidly that the WHO termed this an “infodemic” (a portmanteau of information and pandemic) [14].

Given this landscape, several governments have capitalized on social media itself to engage the public [15,16]. Notably, a growing number of countries—including Australia, Singapore, and the United Kingdom—have used the platform WhatsApp to provide COVID-19 updates (eg, [17]), as has the WHO [18]. Since WhatsApp is the most widely used messaging platform worldwide, these channels allow governments to access large segments of the population [19]. Further, messages can be sent near-instantly, co-opting the very channel used to disseminate false information. Accordingly, platforms like WhatsApp may prove ideal for boosting psychological resilience during this global pandemic.

Despite the potential of messenger-based channels, it remains unclear how effective these are since few health crises have arisen since WhatsApp was introduced in 2009 [20]. To address this gap, we conducted a large-scale survey with the following aims: (1) assess population mental health during the outbreak; (2) replicate findings on how news exposure may predispose an individual to distress; and (3) assess whether receiving updates through official WhatsApp channels can mitigate risk.

Our study was conducted across two months in Singapore, a city state that reported its first case early in the outbreak (January 23, 2020). During the survey period, Singapore experienced a rapid increase in COVID-19 cases, from 138 cases on March 7 (or 24 per million population), to 9125 cases on April 21 (1600 per million population). Consequently, a nationwide lockdown was imposed during the survey period, on April 7. Central to our question, from the outset of the crisis the Singapore government also deployed a WhatsApp subscription service that provided twice-daily updates on the local situation (eg, number of new cases, new infection control measures). At the time of the survey, this had been adopted by approximately 1 in 10 residents [21], providing a natural experiment to evaluate its impact.

## Methods

### Study Design and Population

Across an 8-week span (March 7 to April 21, 2020), we recruited 1145 adults who were aged 21 years and older, and had lived in Singapore for at least 2 years. Participants responded to advertisements placed in Facebook and WhatsApp community groups (eg, groups for residential estates, universities, and workplaces), or to paid Facebook advertisements targeting Singapore-based users who were at least 21 years old. The study was approved by the Yale-NUS College Ethics Review Committee (2020-CERC-001) and was preregistered on ClinicalTrials.gov (NCT04305574). In accordance with the Declaration of Helsinki, all participants gave their consent online prior to completing the survey. Participants received no inconvenience fee for their involvement, and were referred to the Ministry of Health’s COVID-19 website at the end of the study.

### Predictor Variables

Following informed consent, participants completed a 10-minute survey hosted on the online platform Qualtrics [22]. The survey was written at a seventh grade reading level, and was pilot tested for clarity.

As predictors, participants were asked questions pertaining to the following: (1) their exposure to COVID-19 news; (2) their use of the government’s WhatsApp channel; and (3) demographics.

### Exposure to COVID-19 News

To monitor the amount of time spent receiving and discussing COVID-19 news [11], participants were asked to estimate how much time they spent daily getting updates about COVID-19 (eg, searching and reading news) and using social media to discuss or share information. To provide a context for these values, they were also asked whether they spent more, the same, or less time each day being updated with the news and using social media compared to preoutbreak. However, these questions were highly correlated with time estimates and were not included in the statistical analyses.

As the quality of information received has also been found to predict well-being [13], participants were additionally asked about their exposure to COVID-19 rumors. Specifically, participants were shown 5 common myths that have been propagated about COVID-19 (“drinking water frequently will help to prevent infection,” “eating garlic can help to prevent infection,” “the outbreak came about because of people eating bat soup,” “the virus was created in a US lab to affect China’s economy,” and “the virus was created in a Chinese lab as a bioweapon”) [23]. For each claim, participants indicated the following: (1) whether they had heard the claim before; (2) whether they had shared it on social media; and (3) whether they thought it was true [24]. An affirmative response was counted as “1”, and scores were summed across claims to create three subscale scores (corresponding to the hearing, sharing, and belief of claims).

### Use of an Official WhatsApp Channel

As the primary predictors of interest, participants were asked 3 questions pertaining to their use of the government’s WhatsApp channel. Shortly after the first local cases in Singapore (at the end of January 2020), the government began using a subscription-based WhatsApp channel to send out COVID-19 updates in the country’s 4 primary languages (English, Mandarin, Malay, and Tamil). On average, these messages were sent twice a day. The content of these messages included the following: (1) providing an update on confirmed cases (“There are 4 new confirmed cases.”); (2) debunking rumors (eg, “The Prime Minister is not addressing the nation tonight, nor is Singapore locking down.”); (3) disseminating new knowledge (eg, “How does it spread?”); (4) describing infection control measures (eg, “All sports and recreation facilities will be closed.”); (5) describing economic and social support (eg, “Tourism, transport, retail and F&B sectors get additional support.”); or (6) responding to ad hoc events on the ground (eg, “Stay calm; don’t panic buy.”). Messages were written in brief sentences or in point form, with key phrases emphasized in bold (eg, “Observe good personal hygiene, be socially responsible”) and with the use of emoticons (eg, 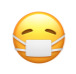). When the study began, this service had 635,000 unique subscribers out of Singapore’s resident population of nearly 6 million individuals (approximately 10%) [21].

To capture WhatsApp usage, participants indicated the following: (1) whether they had used the government’s WhatsApp channel to receive COVID-19 news (yes/no); (2) how likely they were to share or forward messages from the channel (using 4-point scales anchored with “will definitely not forward on” and “will definitely forward on”); and (3) how likely they were to trust a message from this source (using 4-point scales anchored with “do not trust at all” and “trust completely”).

As a basis of comparison, participants also indicated their use of, willingness to share or forward messages from, and trust of 12 possible news sources. These ranged from traditional sources (eg, printed newspapers, radio) to social media (eg, Facebook, YouTube).

### Demographics

As the final category of predictors, participants reported their gender, ethnicity, religion, country of birth, country of citizenship, marital status, education, house type (as a proxy of socioeconomic status), and household size. Based on the survey time stamp, we also recorded the total number of local cases reported to date, and whether the country was locked down at the time of survey completion.

### Outcome Measures

#### Depression, Anxiety, and Stress Scale

As the key outcome measure, participants completed the 21-item Depression, Anxiety, and Stress Scale (DASS-21), a widely used assay of depression (eg, “I felt that I had nothing to look forward to”; Cronbach α=0.90), anxiety (eg, “I felt I was close to panic”; Cronbach α=0.80), and stress symptoms (eg, “I found it hard to wind down”; Cronbach α=0.89) previously validated within this population [25]. During the COVID-19 outbreak, the DASS-21 has also been used to measure psychological symptoms in both community and patient populations [2,26].

#### Expectations and Behavioral Responses Toward the Outbreak

As secondary outcome measures, participants reported their reactions to the COVID-19 outbreak. First, participants were asked about their perceptions regarding the following [27]: (1) how confident they were that the government could control the nationwide spread of COVID-19 (answered using 4-point scales anchored with “not confident at all” and “very confident”); (2) how likely it was that they or someone in their immediate household would be infected with COVID-19 (using 4-point scales anchored with “not at all likely” and “very likely”); and (3) how fearful they were about the COVID-19 situation in Singapore (using 4-point scales anchored with “not scared at all” and “very scared”).

Finally, participants also indicated which of 19 possible measures they had undertaken because of the outbreak (eg, “washed my hands more frequently,” “relied more on online shopping,” “kept a distance from people suspected of recent contact with a COVID-19 case”) [28,29].

### Statistical Analysis

First, to describe participants’ baseline characteristics, survey responses were summarized by total numbers (with percentages) and medians (with interquartile ranges). For the primary analyses, we then ran a series of linear regression models with DASS-21 subscale scores as outcome measures.

In the first model, we sought to replicate findings identifying the receipt of COVID-19 news as a risk factor for psychological symptoms. Correspondingly, this model included the following predictors: (1) two questions pertaining to the time spent processing COVID-19 news (the number of hours spent getting updates each day, and the number of hours spent using social media to discuss or share COVID-19 information), and (2) three questions on COVID-19 rumors (the number of rumors heard, shared, and believed).

For the second model, we explored whether the use of an official WhatsApp channel could be protective, having factored in individual differences in the receipt of COVID-19 news. Here, we repeated the first model with three additional predictors: use of the government’s WhatsApp channel, trust in its messages, and likelihood of sharing its messages (see Multimedia Appendix 1 for Spearman correlations for these predictors).

Finally, we ran two further models to assess the robustness of our findings, controlling for the outbreak situation (Model 3: implementation of a lockdown, and the number of local cases at the point of survey completion) and demographic variables (Model 4: age, gender, ethnicity, religion, marital status, education, house type, household size, and country of birth).

To achieve linearity, the following variables were log-transformed for regression analyses: DASS-21 subscale scores, time spent getting COVID-19 updates, time spent using social media, and the number of local COVID-19 cases. For each model, the type 1 decision-wise error rate was controlled at =0.05, and power calculations showed statistical power at the 0.90 level to detect small effect sizes (f^2^=0.01). Statistical analyses were conducted using SPSS (Version 25; IBM Corp) and R (Version 3.4.0; R Foundation for Statistical Computing).

## Results

### Response Rate

Of 1390 individuals who clicked the survey link, 1145 (82.4%) provided informed consent and participated in the study. As shown in Table 1, the final sample was comparable to the resident population for the following variables: the proportion of Singapore citizens, marital status, and household size (≤10% difference). However, the pool of respondents had a greater representation of females (62.4% versus 51.1%), university graduates (67.2% versus 32.4%), persons aged 21-34 (40.8% versus 25.86%), and persons of no religion (26.6% versus 18.5%) or Christian belief (34.1% versus 18.8%). Conversely, there was a reduced representation of participants who lived in 1- to 3-room public housing flats (7.3% versus 23.7%). Survey respondents were also more likely to be of Chinese ethnicity than persons in the general population (83.4% versus 74.3%).

**Table 1:** Baseline characteristics of survey respondents (N=1145).

Characteristic		Participants, n (%)
**Age group (years)**		
	21-34	467 (40.8)
	35-49	394 (34.4)
	50-64	220 (19.2)
	≥65	64 (5.6)
	Did not answer	0 (0)
**Gender**		
	Female	715 (62.4)
	Male	382 (33.4)
	Did not answer	48 (4.2)
**Ethnicity**		
	Chinese	955 (83.4)
	Indian	53 (4.6)
	Malay	30 (2.6)
	Filipino	18 (1.6)
	Caucasian	14 (1.2)
	Other	27 (2.4)
	Did not answer	48 (4.2)
**Religion**		
	Christianity (Protestant)	390 (34.1)
	No religion	305 (26.6)
	Buddhism	168 (14.7)
	Roman Catholicism	110 (9.6)
	Taoism/Chinese traditional beliefs	45 (3.9)
	Islam	37 (3.2)
	Hinduism	32 (2.8)
	Other	9 (0.8)
	Did not answer	49 (4.3)
**Marital status**		
	Married	613 (53.5)
	Single	319 (27.9)
	Dating	114 (10.0)
	Widowed, separated, or divorced	31 (2.7)
	Did not answer	68 (5.9)
**Educational level**		
	1: Primary school	3 (0.3)
	2: Secondary school	62 (5.4)
	3: Junior college	93 (8.1)
	4: Vocational training	16 (1.4)
	5: Polytechnic or diploma	129 (11.3)
	6: University (undergraduate)	541 (47.2)
	7: University (postgraduate)	229 (20.0)
	Other	4 (0.3)
	Did not answer	68 (5.9)
**House type**		
	1: Public housing flat: 1-2 rooms	9 (0.8)
	2: Public housing flat: 3 rooms	74 (6.5)
	3: Public housing flat: 4 rooms	263 (23.0)
	4: Public housing flat: 5 rooms or executive flats	300 (26.2)
	5: Condominium or private apartments	291 (25.4)
	6: Landed property	127 (11.1)
	Other	13 (1.1)
	Did not answer	68 (5.9)
**Household size**		
	1	52 (4.5)
	2	143 (12.5)
	3	242 (21.1)
	4	327 (28.6)
	≥5	313 (27.3)
	Did not answer	68 (5.9)
**Citizenship**		
	Singapore	964 (84.2)
	Other	133 (11.6)
	Did not answer	48 (4.2)
**Country of birth**		
	Singapore	876 (76.5)
	Other	221 (19.3)
	Did not answer	48 (4.2)
**Years in Singapore**		
	≤20	186 (16.2)
	21-34	420 (36.7)
	35-49	304 (26.6)
	50-64	190 (16.6)
	≥65	45 (3.9)
	Did not answer	0 (0)

### Characterizing the Sample: DASS-21 Symptoms and COVID-19 Responses

**Figure 1:**
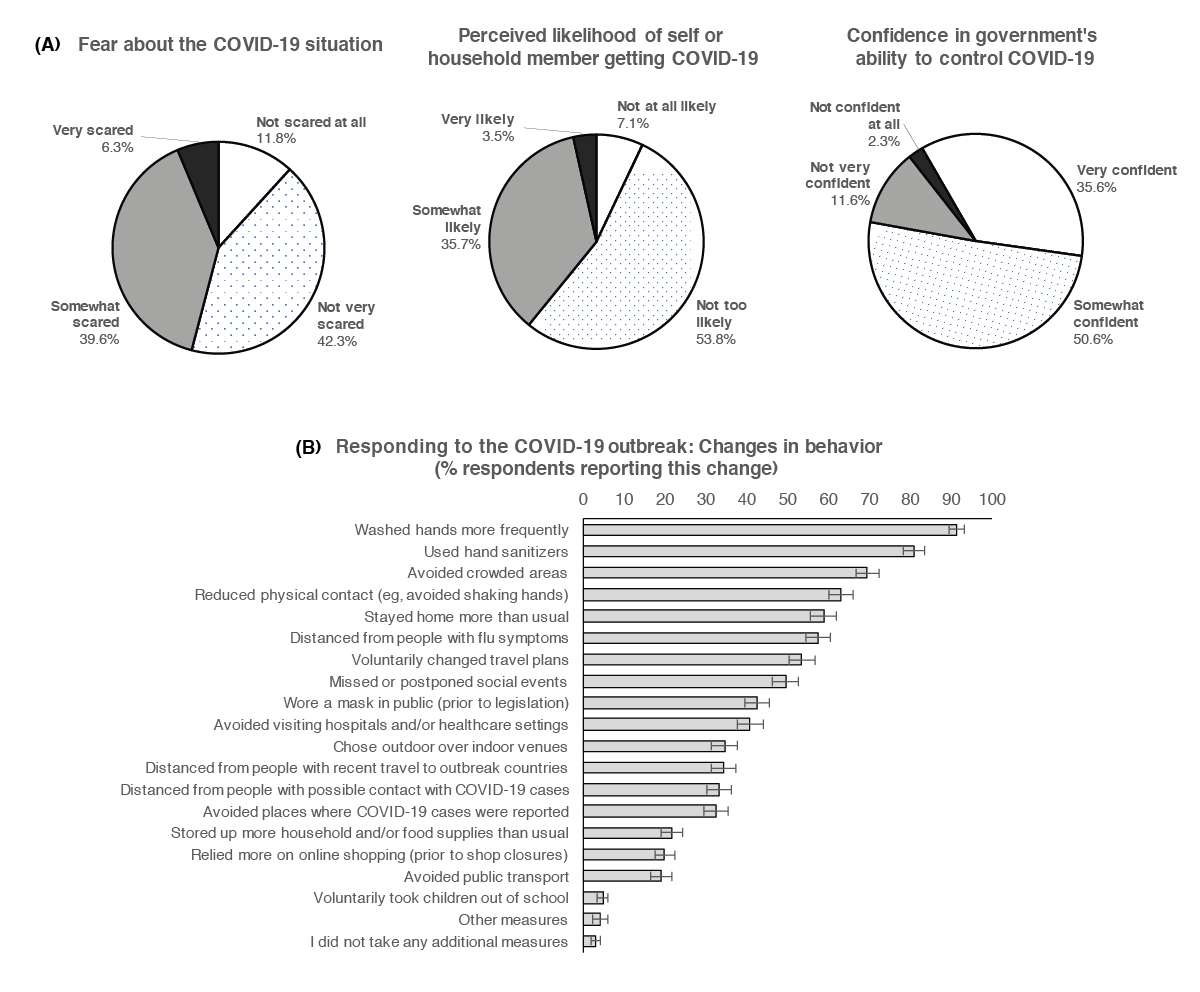
Responses to the COVID-19 outbreak. (A) Participants reported how fearful they were about the COVID-19 situation (top: left-most panel), the likelihood that they or a household member would test positive for COVID-19 (middle panel), and their confidence in the government’s ability to control the outbreak (right-most panel). (B) Additionally, participants also reported the behavioral measures they adopted in response to the outbreak. Vertical lines represent the 95% confidence intervals.

We first sought to characterize our sample participants in terms of their responses to the outbreak. As shown in Figure 1, the average participant was “not very scared” about the COVID-19 situation (median rating of 2, IQR 2-3), deemed it “not too likely” that they or a household member would be infected (median rating of 2, IQR 2-3), and was “somewhat confident” that the government could control the nationwide spread (median rating of 2, IQR 1-2). Most participants (96.9%, 95% CI 95.7%-98.0%) had also voluntarily changed their behavior as a function of the outbreak, adopting measures such as handwashing or avoiding crowds (Figure 1).

**Figure 2:**
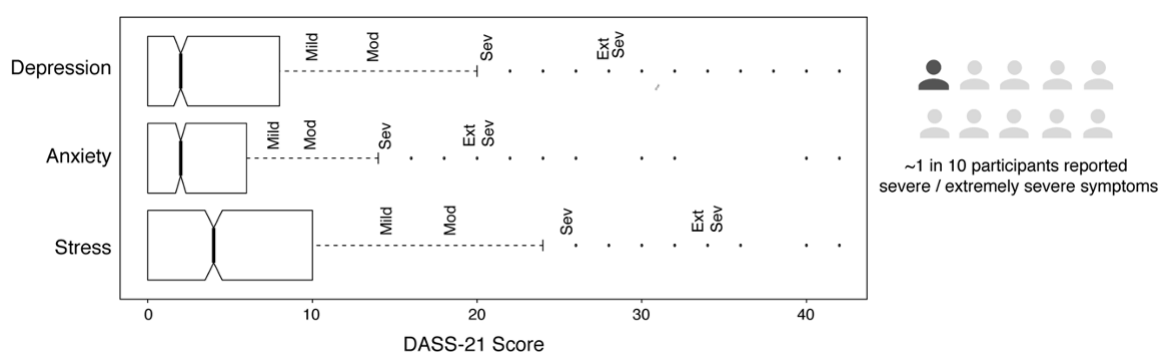
Box plots of participants’ scores on the Depression, Anxiety, and Stress Scale (DASS-21). The width of the box indicates the interquartile range, while the midline depicts the median. Participants’ symptoms were classified as normal, mild, moderate (Mod), severe (Sev), or extremely severe (Ext Sev). Across the three subscales, nearly 1 in 10 participants (7.9%) reported severe or extremely severe symptoms on at least one subscale.

Correspondingly, the majority of participants reported normal levels of depression, anxiety, and stress symptoms on the DASS-21 scale (median scores for the depression, anxiety, and stress subscales were 5.57, 2.0, and 4.0, respectively). Nonetheless, about 1 in 10 participants (7.9%) had severe or extremely severe symptoms on at least one subscale (Figure 2).

### Characterizing the Sample: Use of the Official WhatsApp Channel for COVID-19 News

**Figure 3:**
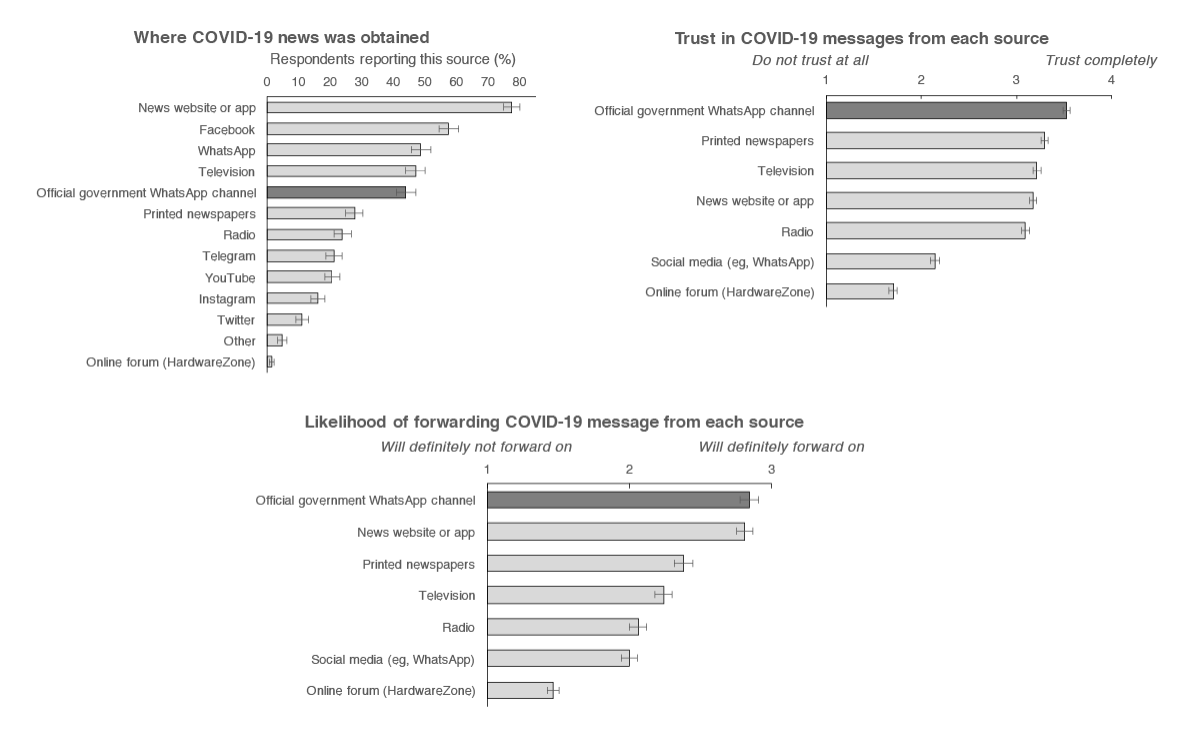
Sources of COVID-19 news. Participants reported where they received COVID-19 news from (top: left panel), the extent to which they trusted each source (top: right panel), and the likelihood of them forwarding messages from each source (bottom panel). Vertical lines represent the 95% confidence intervals.

As shown in Figure 3, nearly 1 in 2 participants (43.9%, 95% CI 40.8%-47%) received updates from the government’s WhatsApp channel. Participants reported trusting the messages received (mean rating of 3.52 of 4, 95% CI 3.48-3.56) and being likely to forward messages from this source (mean rating of 2.84 of 4, 95% CI 2.78-2.90).

For exploratory analyses, we conducted Student *t* tests to examine whether users of the government’s WhatsApp channel differed from nonusers. On the whole, both users and nonusers cut across diverse demographic groups and were largely matched in characteristics (Table 2). However, usage increased as the outbreak progressed (*t*[974]=–3.38, *P*=.01; 95% CI of the difference: 0.17-0.65 weeks). As a group, users also had a higher educational level (*t*[967]=–2.27, *P*=.02; 95% CI 0.03-0.37), and were more likely to be female than nonusers (^2^(1,N=973)=5.03, *P*=.03; 95% CI of the difference in percentage of females: 0.92%-12.9%).

**Table 2:** Participant characteristics as a function of whether they used the official government WhatsApp channel.

Characteristics		Users (n=428)		Nonusers (n=548)		Statistic^a^ (*P* value)	
Survey completion week, mean (SD)		3.4 (2.0)		3.0 (1.8)		–3.38 (.001)^b^	
Age (years), mean (SD)		39.4 (12.1)		39.7 (13.4)		0.36 (.72)	
**Gender^c^, n**						5.03 (.03)^b^	
	Female		295		338		N/A^d^
	Male		133		207		N/A
**Ethnicity^c^, n**						3.57 (.61)	
	Chinese		377		471		N/A
	Indian		23		24		N/A
	Malay		11		14		N/A
	Caucasian		7		6		N/A
	Filipino		4		11		N/A
	Others		0		1		N/A
**Religion^c^, n**						7.40 (.39)	
	Christianity		162		188		N/A
	No religion		113		159		N/A
	Buddhism		61		83		N/A
	Roman Catholicism		39		62		N/A
	Taoism/Chinese traditional beliefs		20		18		N/A
	Hinduism		16		12		N/A
	Islam		13		20		N/A
	Others		4		2		N/A
**Marital status^c^, n**						9.31 (.16)	
	Married		258		291		N/A
	Single		120		169		N/A
	Dating		40		65		N/A
	Widowed, separated, or divorced		10		18		N/A
	Other		0		1		N/A
Educational level, mean (SD)		5.7 (1.3)		5.5 (1.4)		–2.28 (.02)^b^	
House type, mean (SD)		4.1 (1.1)		4.1 (1.2)		–0.07 (.95)	
Household size, mean (SD)		3.7 (1.2)		3.7 (1.2)		0.02 (.98)	
**Citizenship^c^, n**						0.11 (.74)	
	Singapore		374		480		N/A
	Other		54		65		N/A
**Country of birth^c^, n**						0.77 (.38)	
	Singapore		335		439		N/A
	Other		93		106		N/A
Years in Singapore, mean (SD)		34.1 (15.1)		34.6 (16.1)		0.45	

^a^Unless otherwise stated, the test statistic refers to the *t* statistic.

^b^*P*<.05.

^c^Pearson chi-square statistic reported.

^d^N/A: not applicable.

### Predicting Depression, Anxiety, and Stress Symptoms During the COVID-19 Outbreak

#### Depression Symptoms

**Figure 4:**
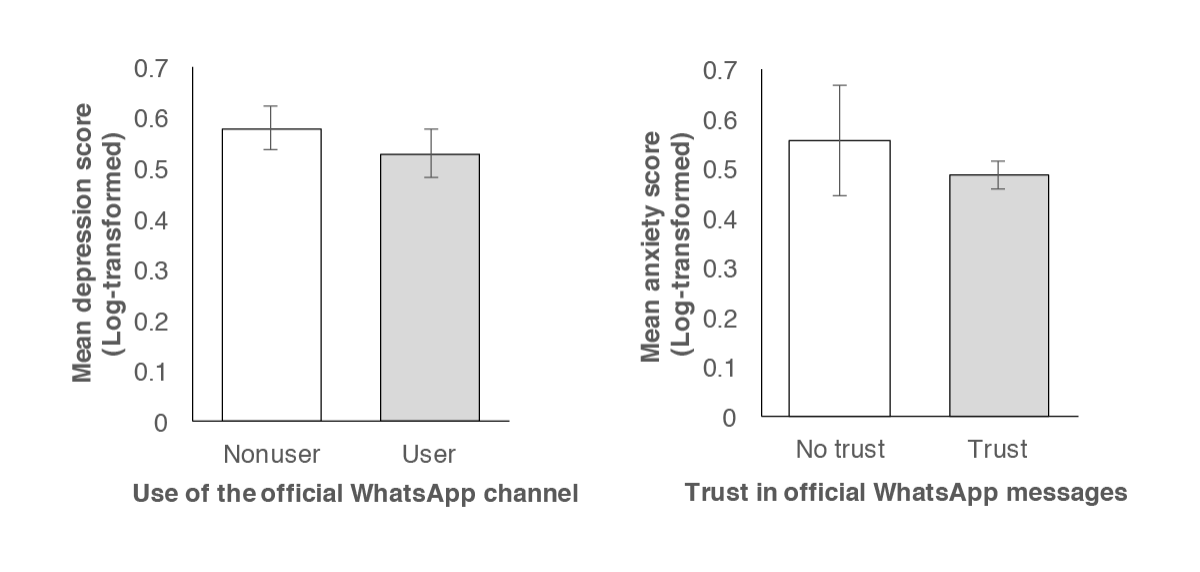
In the left panel, participants’ depression scores on the Depression, Anxiety, and Stress Scale (DASS-21) have been plotted as a function of their use of the government’s WhatsApp channel for COVID-19 information. In the right panel, corresponding anxiety scores have been plotted as a function of trust in the channel’s messages. Vertical lines represent 95% confidence intervals.

The following sections address the primary research question, highlighting characteristics that predict depression, anxiety, and stress symptoms on the DASS-21 scale. In terms of depression, the amount of time participants spent receiving COVID-19 updates robustly predicted well-being, with increased time spent linked to increased depression scores (Models 1-4; Table 3). Having controlled for exposure to COVID-19 news, however, use of the government’s WhatsApp channel emerged as a protective factor (Model 2). Namely, channel users reported lower depression scores than nonusers (Figure 4; *b*=–0.07, *t*[>863]=–2.04, *P*=.04). This association persisted after situational and demographic factors were controlled for in Models 3 and 4, respectively. Finally, the following situational and demographic variables also predicted depression scores (*P*<.05): the number of local COVID-19 cases to date (Models 3 and 4), age (Model 4), and marital status (Model 4).

**Table 3:** Predicting depression symptoms during the COVID-19 outbreak.

Dependent variable: depression symptoms (DASS-21)^a,b^		Model 1		Model 2		Model 3		Model 4	
Time spent getting COVID-19 updates (hours per day)^b^		0.448^c^ (0.239 to 0.656)		0.454^c^ (0.246 to 0.662)		0.377^c^ (0.169 to 0.585)		0.389^c^ (0.183 to 0.595)	
Time spent using social media to discuss or share COVID-19 information (hours per day)^b^		0.072 (–0.134 to 0.279)		0.073 (–0.134 to 0.280)		0.033 (–0.171 to 0.238)		0.055 (–0.147 to 0.257)	
Number of COVID-19 rumors heard		0.009 (–0.015 to 0.034)		0.012 (–0.012 to 0.037)		0.006 (–0.019 to 0.030)		0.010 (–0.014 to 0.034)	
Number of COVID-19 rumors shared		0.042 (–0.010 to 0.094)		0.040 (–0.012 to 0.092)		0.038 (–0.013 to 0.089)		0.043 (–0.007 to 0.094)	
Number of COVID-19 rumors believed		0.037 (–0.016 to 0.090)		0.035 (–0.018 to 0.088)		0.039 (–0.013 to 0.091)		0.033 (–0.020 to 0.085)	
Use of government’s WhatsApp channel (reference: nonuser)		N/A^d^		–0.070^e^ (–0.137 to –0.003)		–0.086^e^ (–0.153 to –0.019)		–0.083^e^ (–0.149 to –0.017)	
Trust in government’s WhatsApp messages		N/A		–0.032 (–0.087 to 0.024)		–0.026 (–0.061 to 0.023)		–0.017 (–0.072 to 0.038)	
Likelihood of sharing government’s WhatsApp messages		N/A		0.015 (–0.020 to 0.050)		0.014 (–0.021 to 0.049)		0.014 (–0.020 0.049)	
Lockdown (reference: no lockdown)		N/A		N/A		–0.077 (–0.266 to 0.111)		–0.065 (–0.254 to 0.125)	
Local COVID-19 cases to date^b^		N/A		N/A		0.224^c^ (0.064 to 0.384)		0.195^e^ (0.036 to 0.355)	
Age in years		N/A		N/A		N/A		–0.004^c^ (–0.007 to –0.001)	
Gender (reference: female)		N/A		N/A		N/A		–0.006 (–0.072 to 0.059)	
**Ethnicity (reference: Chinese)**									
	Indian		N/A		N/A		N/A		–0.028 (–0.277 to 0.221)
	Malay		N/A		N/A		N/A		–0.316 (–0.694 to 0.062)
	Filipino		N/A		N/A		N/A		0.194 (–0.126 to 0.514)
	Caucasian		N/A		N/A		N/A		0.009 (–0.255 to 0.274)
	Other		N/A		N/A		N/A		–0.003 (–0.909 to 0.902)
**Religion (reference: no religion)**									
	Christianity		N/A		N/A		N/A		–0.018 (–0.097 to 0.061)
	Buddhism		N/A		N/A		N/A		0.059 (–0.041 to 0.159)
	Roman Catholicism		N/A		N/A		N/A		<.001 (–0.118 to 0.118)
	Taoism/Chinese traditional beliefs		N/A		N/A		N/A		0.070 (–0.090 to 0.230)
	Islam		N/A		N/A		N/A		0.306 (–0.068 to 0.680)
	Hinduism		N/A		N/A		N/A		–0.124 (–0.433 to 0.185)
**Marital status (reference: single)**									
	Married		N/A		N/A		N/A		–0.124^c^ (–0.204 to –0.045)
	Dating		N/A		N/A		N/A		0.051 (–0.060 to 0.162)
	Widowed, separated, or divorced		N/A		N/A		N/A		–0.114 (–0.310 to 0.082)
Educational level		N/A		N/A		N/A		–0.007 (–0.031 to 0.018)	
House type		N/A		N/A		N/A		0.018 (–0.011 to .047)	
Household size		N/A		N/A		N/A		–0.027 (–0.056 to 0.002)	
Country of birth (reference: not Singapore)		N/A		N/A		N/A		–0.011 (–0.097 to 0.076)	
*R* ^2^		.06		.06		.09		.16	

^a^Dependent variable: depression subscale scores from the 21-item Depression, Anxiety, and Stress Scale (DASS-21). Data reported as beta estimates (95% CI).

^b^Log-transformed.

^c^*P*<.01.

^d^N/A: not applicable.

^e^*P*<.05.

#### Anxiety Symptoms

As shown in Table 4, anxiety symptoms were likewise predicted by exposure to COVID-19 news in a robust manner. Across all 4 models, increased anxiety scores were associated with increased time spent obtaining COVID-19 updates and increased exposure to, sharing of, and belief of COVID-19 rumors. After controlling for these variables (Model 2), trust in the government’s WhatsApp channel emerged as a protective factor, with increased trust predicting lower anxiety scores (Figure 4; *b*=–0.05, *t*[863]=–2.13, *P*=.03). This relationship held when the model was adjusted for situational and demographic variables (Model 3 and 4), with the following variables emerging as additional predictors of anxiety scores (*P*<.05): being in a lockdown (Models 3 and 4), the number of local cases to date (Models 3 and 4), and age (Model 4).

**Table 4:** Predicting anxiety symptoms during the COVID-19 outbreak.

Dependent variable: anxiety symptoms (DASS-21)^a,b^		Model 1	Model 2	Model 3	Model 4
Time spent getting COVID-19 updates (hours per day)^b^		0.278^c^ (0.100 to 0.456)	0.282^c^ (0.104 to 0.459)	0.262^c^ (0.083 to 0.442)	0.283^c^ (0.104 to 0.463)
Time spent using social media to discuss or share COVID-19 information (hours per day)^b^		0.010 (–0.166 to 0.187)	0.003 (–0.174 to 0.180)	–0.006 (–0.183 to 0.171)	0.002 (–0.175 to 0.178)
Number of COVID-19 rumors heard		0.032^c^ (0.011 to 0.053)	0.032^c^ (0.011 to 0.053)	0.030^c^ (0.009 to 0.051)	0.033^c^ (0.012 to 0.054)
Number of COVID-19 rumors shared		0.083^c^ (0.039 to 0.127)	0.080^c^ (0.036 to 0.125)	0.080^c^ (0.036 to 0.125)	0.082^c^ (0.038 to 0.126)
Number of COVID-19 rumors believed		0.068^c^ (0.023 to 0.113)	0.069^c^ (0.023 to 0.114)	0.067^c^ (0.022 to 0.113)	0.062^c^ (0.016 to 0.107)
Use of government’s WhatsApp channel (reference: nonuser)		N/A^d^	0.023 (–0.034 to 0.081)	0.021 (–0.037 to 0.078)	0.025 (–0.032 to 0.082)
Trust in government’s WhatsApp messages		N/A	–0.051^e^ (–0.099 to –0.004)	–0.050^e^ (–0.097 to 0.003)	–0.054^e^ (–0.101 to –0.006)
Likelihood of sharing government’s WhatsApp messages		N/A	0.017 (–0.013 to 0.047)	0.017 (–0.013 to 0.047)	0.015 (–0.015 to 0.046)
Lockdown (reference: no lockdown*)*		N/A	N/A	–0.190^e^ (–0.353 to –0.027)	–0.186^e^ (–0.352 to –0.021)
Local COVID-19 cases to date^b^		N/A	N/A	0.165^e^ (0.027 to 0.303)	0.151^e^ (0.012 to 0.290)
Age in years		N/A	N/A	N/A	–0.004^c^ (–0.006 to –0.001)
Gender (reference: female)		N/A	N/A	N/A	–0.036 (–0.093 to 0.022)
**Ethnicity (reference: Chinese)**					
	Indian	N/A	N/A	N/A	0.135 (–0.083 to 0.352)
	Malay	N/A	N/A	N/A	–0.038 (–0.367 to 0.292)
	Filipino	N/A	N/A	N/A	0.057 (–0.210 to 0.324)
	Caucasian	N/A	N/A	N/A	–0.101 (–0.332 to 0.131)
	Other	N/A	N/A	N/A	0.171 (–0.619 to 0.961)
**Religion (reference: no religion)**					
	Christianity	N/A	N/A	N/A	–0.031 (–0.100 to 0.038)
	Buddhism	N/A	N/A	N/A	0.050 (–0.037 to 0.138)
	Roman Catholicism	N/A	N/A	N/A	0.033 (–0.070 to 0.135)
	Taoism/Chinese traditional beliefs	N/A	N/A	N/A	0.035 (–0.105 to 0.175)
	Islam	N/A	N/A	N/A	0.122 (–0.205 to 0.448)
	Hinduism	N/A	N/A	N/A	–0.260 (–0.530 to 0.009)
**Marital status (reference: single)**					
	Married	N/A	N/A	N/A	–0.063 (–0.132 to 0.006)
	Dating	N/A	N/A	N/A	–0.009 (–0.106 to 0.088)
	Widowed, separated, or divorced	N/A	N/A	N/A	–0.116 (–0.287 to 0.056)
Educational level		N/A	N/A	N/A	–0.012 (–0.034 to 0.009)
House type		N/A	N/A	N/A	0.017 (–0.008 to 0.042)
Household size		N/A	N/A	N/A	–0.009 (–0.034 to 0.017)
Country of birth (reference: not Singapore)		N/A	N/A	N/A	–0.041 (–0.117 to 0.034)
*R* ^2^		.06	.07	.08	.12

^a^Dependent variable: anxiety subscale scores from the 21-item Depression, Anxiety, and Stress Scale (DASS-21). Data reported as beta estimates (95% CI).

^b^Log-transformed.

^c^*P*<.01.

^d^N/A: not applicable.

^e^*P*<.05.

#### Stress Symptoms

Finally, Table 5 again highlights how the amount of time spent getting updates predicted stress symptoms (Models 1-4). However, there was no significant predictor related to use of the official WhatsApp channel (Models 2-4). Nonetheless, the following variables emerged as significant predictors of stress: being in a lockdown (Models 3-4), the number of local cases to date (Models 3-4), age (Model 4), and gender (Model 4).

**Table 5:** Predicting stress symptoms during the COVID-19 outbreak.

Dependent variable: stress symptoms (DASS-21)^a,b^		Model 1	Model 2	Model 3	Model 4
Time spent getting COVID-19 updates (hours per day)^b^		0.405^c^ (0.200 to 0.611)	0.411^c^ (0.206 to 0.617)	0.353^c^ (0.147 to 0.559)	0.385^c^ (0.180 to 0.590)
Time spent using social media to discuss or share COVID-19 information (hours per day)^b^		–0.058 (–0.262 to 0.145)	–0.061 (–0.266 to 0.143)	–0.091 (–0.294 to 0.113)	–0.057 (–0.258 to 0.144)
Number of COVID-19 rumors heard		0.020 (–0.004 to 0.044)	0.020 (–0.004 to 0.044)	0.015 (–0.009 to 0.039)	0.019 (–0.005 to 0.043)
Number of COVID-19 rumors shared		0.043 (–0.008 to 0.094)	0.041 (–0.010 to 0.092)	0.040 (–0.010 to 0.091)	0.051^d^ (0.001 to 0.101)
Number of COVID-19 rumors believed		0.031 (–0.021 to 0.084)	0.032 (–0.020 to 0.085)	0.033 (0.019 to 0.085)	0.026 (–0.026 to 0.078)
Use of government’s WhatsApp channel (reference: nonuser)		N/A^e^	0.017 (–0.049 to 0.083)	0.006 (–0.060 to 0.072)	0.004 (–0.062 to 0.069)
Trust in government’s WhatsApp messages		N/A	–0.048 (–0.103 to 0.007)	–0.044 (–0.098 to 0.011)	–0.048 (–0.102 to 0.007)
Likelihood of sharing government’s WhatsApp messages		N/A	0.007 (–0.028 to 0.041)	0.006 (–0.029 to 0.041)	0.004 (–0.031 to 0.038)
Lockdown (reference: no lockdown)		N/A	N/A	–0.190^d^ (–0.378 to –0.003)	–0.204^d^ (–0.393 to –0.016)
Local COVID-19 cases to date^b^		N/A	N/A	0.254^c^ (0.095 to 0.413**)**	0.249^c^ (0.090 to 0.407)
Age in years		N/A	N/A	N/A	–0.005^c^ (–0.008 to –0.002)
Gender (reference: female)		N/A	N/A	N/A	–0.084^d^ (–0.150 to –0.019)
**Ethnicity (reference: Chinese)**					
	Indian	N/A	N/A	N/A	–0.056 (–0.303 to 0.192)
	Malay	N/A	N/A	N/A	–0.290 (–0.666 to 0.086)
	Filipino	N/A	N/A	N/A	–0.017 (–0.335 to 0.301)
	Caucasian	N/A	N/A	N/A	–0.036 (–0.299 to 0.227)
	Other	N/A	N/A	N/A	0.144 (–0.756 to 1.045)
**Religion (reference: no religion)**					
	Christianity	N/A	N/A	N/A	–0.038 (–0.117 to 0.040)
	Buddhism	N/A	N/A	N/A	0.042 (–0.057 to 0.142)
	Roman Catholicism	N/A	N/A	N/A	0.050 (–0.067 to –0.167)
	Taoism/Chinese traditional beliefs	N/A	N/A	N/A	0.069 (–0.090 to 0.229)
	Islam	N/A	N/A	N/A	0.311 (–0.061 to 0.683)
	Hinduism	N/A	N/A	N/A	–0.155 (–0.462 to 0.152)
**Marital status (reference: single)**					
	Married	N/A	N/A	N/A	–0.044 (–0.123 to 0.035)
	Dating	N/A	N/A	N/A	0.084 (–0.027 to 0.195)
	Widowed, separated, or divorced	N/A	N/A	N/A	–0.179 (–0.374 to 0.016)
Educational level		N/A	N/A	N/A	0.009 (–0.015 to 0.033)
House type		N/A	N/A	N/A	0.019 (–0.010 to 0.047)
Household size		N/A	N/A	N/A	–0.010 (–0.039 to 0.019)
Country of birth (reference: not Singapore)		N/A	N/A	N/A	–0.019 (–0.105 to 0.067)
*R* ^2^		.04	.04	.06	.12

^a^Dependent variable: stress subscale scores from the 21-item Depression, Anxiety, and Stress Scale (DASS-21). Data reported as beta estimates (95% CI).

^b^Log-transformed

^c^*P*<.01.

^d^*P*<.05.

^e^N/A: not applicable.

## Discussion

In the COVID-19 pandemic, digital technology offers novel solutions to disseminate public health messages [15]. Prior to this study, however, there were few studies evaluating these solutions. Accordingly, we systematically examined the governmental use of WhatsApp to provide COVID-19 updates.

As the first aim of our study, we sought to replicate previous findings that had linked psychological distress to COVID-19 news exposure [11]. This pattern also emerged in our data set, such that participants who spent more time getting updates were more likely to display depression, anxiety, and stress symptoms. Similar findings have also been reported in other crises (eg, when Hong Kong experienced social unrest, the amount of time an individual spent dwelling on sociopolitical news predicted symptoms of depression and posttraumatic stress disorder [30]). These findings raise a paradox: although transparency promotes resilience [2] and information seeking is encouraged (eg, [31]), a longer amount of time spent doing so is a marker for poor psychological health.

Construing exposure to COVID-19 news as a modifiable risk factor, one possible way to mitigate risk may be through spreading official advisories via social media. In particular, messenger-based platforms allow a government’s advisories to reach a large number of people near-instantly, minimizing the need for individuals to search for updates themselves or to sift through misinformation. In support of this case, we observed that having controlled for news exposure, subscription to a government’s WhatsApp channel was associated with fewer depression symptoms. Similarly, increased trust in official WhatsApp messages was associated with decreased anxiety. Both these associations were robust and were observed even after situational and demographic characteristics had been adjusted for.

Although preliminary, it is promising that using an official WhatsApp channel may boost psychological resilience. Further research is needed to identify what mechanisms may underlie these findings. One possibility is that WhatsApp allows fast message transmission, curbing the spread of rumors that may provoke anxiety. Alternatively, the use of an official WhatsApp channel may boost trust in the institution, a factor that has been linked with reduced anxiety in times of crisis (eg, the nuclear disaster in Fukushima) [32,33]. Although these accounts are consistent with anxiety being characterized by worry and the threat of danger (such that increased trust in official messages can allay fears) [34], it is less clear why use of the WhatsApp channel was linked to attenuated depression symptoms.

Mechanisms notwithstanding, another important observation was that the official WhatsApp service was adopted by persons of diverse demographic backgrounds. This stands in contrast to traditional findings of certain groups being more technologically savvy (eg, young adults) [35], and likely reflects the broad appeal of WhatsApp (which reports over 2 billion users across 180 countries [36]). Aside from its take-up, participants also reported being willing to forward COVID-19 messages from this source. Consequently, the features of an official WhatsApp service may render it suitable for addressing false information during the current “infodemic.”

In presenting these findings, we note several limitations of our study. First, we used the approach of large-scale epidemiological surveys, examining the real-life usage of an official WhatsApp channel. Although this design is appropriate for new areas of research, the cross-sectional design precludes strong conclusions about causality. Accordingly, future studies should consider randomized controlled trials (eg, by offering subscription to a random sample of a population when a new WhatsApp service is rolled out). Second, we chose to study WhatsApp because of its large worldwide subscription base and because it is the most widely used messenger platform in official deployment (eg, by the WHO and individual governments). In making this choice, however, we were unable to draw conclusions about other messenger-based platforms (eg, Telegram). Finally, we caution the reader about several features of our study that can limit generalizability. Although we sampled from a wide array of demographic groups, our final set of respondents was not representative of the general population. Correspondingly, further research is needed to examine whether our findings can generalize to populations we have undersampled (eg, participants >65 years of age who may be less likely to respond to online recruitment methods). In addition, we note that the context for COVID-19 research is changing rapidly across countries as well as time (eg, number of COVID-19 cases, progress of vaccine research, government-level implementation of infection control measures). Notably, participants in our sample had high trust in official WhatsApp messages and displayed confidence that the government could manage the spread of COVID-19. Moving forward, future research will need to examine the extent to which these elements are crucial to WhatsApp’s effectiveness. To this end, we have provided detailed documentation on the context of our research, allowing the reader to make informed judgments regarding generalizability.

In conclusion, the COVID-19 pandemic has developed against a backdrop of innovative solutions for widespread communication. In unprecedented times, these solutions have the potential to boost psychological resilience; as we reported in this study, one such solution is the use of WhatsApp by a government to disseminate local updates. Given the promising nature of our findings, we encourage governments and transgovernmental bodies to explore these digital technologies. We further encourage researchers to empirically evaluate the impact of these solutions.

## Appendix

Multimedia Appendix 1 Means, standard deviation, and Spearman rho for predictors related to official WhatsApp use.

## Abbreviations

WHO: World Health Organization
