# Supplementary material for: The Relation Between Official WhatsApp-Distributed COVID-19 News Exposure and Psychological Symptoms: Cross-Sectional Survey Study
Source: J Med Internet Res. 2020 Sep 25;22(9):e22142. doi: 10.2196/22142 (PMC7527032; doi:10.2196/22142)
Supplement: Multimedia Appendix 1 [file jmir_v22i9e22142_app1.docx]

**Appendix 1**

*Table S1.* Means, standard deviation, and Spearman’s rho for predictors related to official WhatsApp use

|  | M | SD | 2. Trust in WhatsApp messages | 3. Likelihood of sharing WhatsApp messages |
| --- | --- | --- | --- | --- |
| 1. Use of government’s WhatsApp channel | .44 | .50 | .23 | .28 |
| 2. Trust in WhatsApp messages | 3.52 | .63 |  | .38 |
| 3. Likelihood of sharing WhatsApp messages | 2.84 | 1.01 |  |  |
